# Understanding and Prevention of “Therapy-” Induced Dyskinesias

**DOI:** 10.1155/2012/640815

**Published:** 2012-05-23

**Authors:** Iciar Aviles-Olmos, Zinovia Kefalopoulou, Thomas Foltynie

**Affiliations:** Unit of Functional Neurosurgery Sobell Department of Motor Neuroscience and Movement Disorders, UCL Institute of Neurology, London WC1N3BG, UK

## Abstract

L-dopa is the most effective, currently available treatment for Parkinson's disease (PD), but it leads to the development of involuntary movements known as L-dopa-induced dyskinesia (LID) in the majority of patients after long-term use. Both gene and cell therapy approaches are the subject of multiple ongoing studies as potential ways of relieving symptoms of PD without the complication of dyskinesia. However, the spectre of dyskinesia in the absence of L-dopa, the so-called “off-phase” or graft-induced dyskinesia (GID), remains a major obstacle particularly in the further development of cell therapy in PD, but it is also a concern for proponents of gene therapy approaches. LID results from nonphysiological dopamine release, supersensitivity of dopamine receptors, and consequent abnormal signalling through mechanisms of synaptic plasticity. Restoration of physiological circuitry within the basal ganglia loops is ultimately the aim of all cell and gene therapy approaches but each using distinctive strategies and accompanied by risks of exacerbation of LID or development of “off-phase”/GID. In this paper we discuss the details of what is understood regarding the development of dyskinesias with relevance to cell and gene therapy and potential strategies to minimize their occurrence.

## 1. Introduction

L-dopa is the most effective treatment for Parkinson's disease currently available and for many patients can provide effective relief of symptoms for many years after diagnosis. In most patients, L-dopa treatment leads to a “honeymoon” period during which the motor symptoms are well controlled. However, after 5 years of treatment, approximately 40% of patients will develop fluctuations in symptom control in response to the drug, as well as involuntary movements known as “L-dopa-induced dyskinesias” (LID) [1]. These complications affect as many as 89% of PD patients after 10 years of L-dopa treatment [2]. LID can be seen during “peak dose” periods, during “off” medication periods or in a “biphasic” pattern as L-dopa levels rise and fall following oral intake. For this reason, other strategies have been developed to try and restore normal function of the basal ganglia circuitry. Deep brain stimulation (DBS) of the subthalamic nucleus (STN) or globus pallidus pars interna (GPi) have both been shown to be highly successful ways of controlling symptoms of PD. Dyskinesia is generally reduced following STN DBS as a result of reduction in L-dopa dose. GPi DBS is a highly effective way of reducing LID, but many patients remain reliant on frequent high doses of L-dopa to maintain control of PD symptoms [3, 4].

To improve upon the limitations of currently available therapies, studies are being performed to assess the role of either gene therapy or cell therapy to provide PD symptom relief without the complication of dyskinesia. Cell therapy trials have been seriously hindered by reports of dyskinesia occurring in the absence of L-dopa—the so-called “off-phase” or graft-induced dyskinesia (GID) [5, 6]. There are also theoretical concerns that such “off-phase” dyskinesias might limit the ability of gene therapy to lead to an effective PD therapy. As a prelude to discussing the origin of “therapy-” induced dyskinesia, and strategies to minimize or control them, a discussion of our current understanding of LID is required.

## 2. Origin of LID

Risk factors for the development of LID include younger age [7, 8], dose of L-dopa [9], pattern of L-dopa administration [9–12], and stage of disease [13–15]. The neural mechanisms that underlie LID in PD are not completely understood; however, the study of basal ganglia anatomy and physiology in the normal and dopamine-depleted states has been of great help. In PD, the degeneration of the dopaminergic neurons of the substantia nigra compacta (SNc) compromises the equilibrium between the direct (D1 receptor) and indirect (D2 receptor) pathways resulting in abnormal GPi hyperactivity. Initially, the clinical features of PD were thought to follow simple increases in the “rate” of activity of the GPi, which through inhibition of the motor thalamus acted as a brake to activity in the supplementary motor area. It was further considered that excessive levodopa stimulation might induce dyskinesia by reduction of the inhibition of thalamocortical neurons resulting in an overactivity of motor cortical areas [16].

This model, is however, inconsistent with several clinical and experimental findings. During LID in the nonhuman primate model of PD, there is decreased rather than increased metabolic activity in the ventral anterior (VA) and ventrolateral (VL) thalamic nuclei, regions of the thalamus that receive input from the GPi [17]. Furthermore, among patients with PD and LID, creation of a lesion within the GPi (pallidotomy) is associated with a reduction in LID rather than a deterioration in LID that would have been predicted by the previously described “rate model” [18].

The pathophysiological changes that underlie the development of LID must therefore be far more complex. Recordings taken from PD patients undergoing DBS surgery have revealed that during periods of LID there is an increase in 4–10 Hz activity in the contralateral STN, suggesting that there is an abnormal pattern of oscillatory activity throughout the basal ganglia [19]. The cause of this oscillatory activity is likely to be multifactorial involving both pre and post synaptic components.

### 2.1. Dysfunctional Dopamine Release

The surviving dopaminergic neurons in the progressively denervated striatum, sprout branches that successfully compensate for the neurodegenerative process until *∼*60% of neurons, are lost. Until this point, administration of L-dopa does not alter the concentration of striatal dopamine, but beyond the 60% deficit, the concentration of dopamine in the striatum increases 3-fold after L-dopa administration [20]. While L-dopa administration continues to enhance dopamine synthesis and release by the surviving dopaminergic neurons, L-dopa is also decarboxylated and released as dopamine by serotonergic terminals, noradrenergic neurons, striatal capillaries, and nonaminergic striatal interneurons [20–23]. These terminals do not store and release dopamine in a regulated way, thus leading to nonphysiological dopamine receptor stimulation [24].

The role of serotonergic neurons in the development of LID has been the subject of particular study. In rats with 6-hydroxy dopamine lesions, approximately 80% of the peak dopamine (DA) efflux measured in the striatum following the administration of L-dopa originates from serotonergic neurons [25–28].This nonphysiological DA release is highly dyskinesigenic; indeed recent evidence shows that dyskinetic rodents have increased numbers of serotonergic terminals and sprouting of serotonin axon varicosities stimulated by L-dopa exposure, leading to larger swings of extracellular DA release [29].

### 2.2. Dopamine Receptor Supersensitivity

Under conditions of chronic denervation, dopamine receptors develop supersensitivity, involving an increased expression of receptors on the postsynaptic membrane of medium spiny neurons [30, 31]. D1 receptor supersensitivity has been shown to have a direct relationship with LID severity [32]. Persistent stimulation of dopamine receptors normally leads to their desensitisation and induces receptor internalisation, and it is hypothesised that, in LID, this desensitisation and internalisation process is impaired [33–35]. In a rodent model of LID, it seems that D1 receptors become “anchored” on the plasma membrane of medium spiny neurons due to crosstalk with D3 receptors following chronic administration of L-dopa [34]. Consistent with this it has been shown that the use of a D3 antagonist restores normal levels of D1 receptors on the plasma membrane and has been associated with reduction in LID in both the rodent and primate models [36, 37]. However, it is clear that LID does not occur solely as a result of abnormal D1 receptor expression or sensitivity alone since D2 selective agonists can also provoke dyskinesia [38].

### 2.3. Synaptic Plasticity

Physiological dopaminergic input from nigrostriatal neurons onto the striatal medium spiny neurons plays an important role in the potentiation and depotentiation of synapses of the corticostriatal pathway. Repetitive stimulation can cause either a long-lasting increase in synaptic strength known as long-term potentiation (LTP), or an enduring decrease known as long-term depression (LTD), a phenomenon known as synaptic plasticity [39, 40]. It is this process that allows deletion of unwanted or unnecessary connections and strengthening of desirable motor programs.

Disruption of normal synaptic plasticity is strongly linked to the appearance of dyskinesia [41–45]. It is hypothesized that the disrupted motor control underlying dyskinesia is attributable to specific changes occurring along the dopamine D1 receptor/protein kinase A/dopamine and cyclic AMP-regulated phosphoprotein-32 (D1/PKA/DARPP-32) intracellular signalling pathway leading to the loss of synaptic depotentiation at corticostriatal synapses and to the development of nonphysiological motor circuits within the basal ganglia [39].

L-dopa can restore normal synaptic plasticity among individuals free of dyskinesia, but not when dyskinesias are already developed [46]. It has been proposed that patients with LID have lost the ability to depotentiate synapses normally, that is, they have lost the mechanism that underlies “synaptic forgetting,” resulting in pathological storage of information that would normally be erased, leading to the development of abnormal motor patterns, that is, LID [42, 47]. Possible consequences of this process include increased phosphorylation of N-methyl-D-aspartate (NMDA) and alpha-amino-3-hydroxy-5-methyl-4-isoxazole propionic acid (AMPA) receptor subunits [48] and increased striatal dynorphin mRNA levels [49]. Amelioration of abnormal glutamatergic NMDA transmission likely explains the beneficial effects of Amantadine [50–52] on reversal of LID.

## 3. Graft-Induced Dyskinesia

Graft-induced dyskinesia (GID) was first brought to widespread attention following the publication of the randomised sham-surgery-controlled trials of fetal dopaminergic cell transplantation [5, 6]. GIDs are characterized by the presence of both hyperkinesias and dystonic postures occurring in the “off phase,” generally considered to be greater than 12 hours following the last dose of L-dopa. In the Freed trial, “off phase” GID was seen in 5/33 (15%) of transplanted patients, more than 1 year after transplant [5]. The second sham-surgery-randomised-controlled trial (Olanow trial) reported the presence of “off-phase” GID in 13/23 (56.5%) of the grafted patients, 6–12 months after transplantation [6]. In 3 of the patients from the Olanow trial the GIDs were disabling enough to require further neurosurgical intervention [6]. In contrast to the worrying appearance of “off-phase” GID, neither trial reported any increase in L-dopa-induced or “on-phase” dyskinesia.

Patients receiving open label transplants that predated the Freed and Olanow trials had also experienced GID [53–55] but these had caused only mild to moderate disability to the patients even with follow-up extending to 11 years [55]. Speculation regarding the origin of “off-phase” dyskinesias has included the possibility of excessive dopamine production by the grafts, dopamine receptor supersensitivity away from “islands” of grafted cells, individual factors related to the patient and their PD phenotype, a relationship with immune rejection, or contamination of the grafts with cells of a nondopaminergic phenotype.

### 3.1. Excess Dopamine Release?

Initial hypotheses were that GID occurred due to excessive dopamine release by the grafts. This has not, however, been supported by functional imaging of the patients in the Olanow trial or the Hagell patient series [55–57]. Dyskinesia severity was not related to the magnitude of graft-derived dopaminergic reinnervation, as judged by 18F-dopa positron emission tomography (PET), indicating that “off-phase” dyskinesias probably do not result from excessive growth of grafted dopaminergic neurons. Furthermore, the severity of GID was not correlated with improvement in the “off” UPDRS motor scores [55, 58]. The fact that GIDs have not been seen in patients without any benefit from their transplants suggests that a functional graft is necessary for GID development although their appearance does not simply relate to excess dopamine release.

### 3.2. Imbalanced Dopamine Release?

It was further proposed that islands of excessive dopaminergic activity might relate to GID [59]. Indeed the patients with the best functional outcome after transplantation exhibited no dopaminergic denervation in areas outside the grafted areas, either preoperatively or at 1 or 2 years postoperatively. Comparing PET signal among patients with and without GID, there was a greater increase of putaminal 18F-Dopa uptake seen in the posterodorsal zone of GID patients. However, the GID group also showed a relative increase ventrally not seen at all in the GID-negative patients suggesting that unbalanced increases in dopaminergic function might complicate the outcome of neuronal transplantation for parkinsonism. The implantation of dopamine cells into the ventrocaudal putamen, may also contribute to the unusual distribution of GID, in which the face, neck, and arms tend to be the most clinically involved. These data are corroborated by animal models showing that dyskinesias occur following transplantation of cells to a particular prodyskinetic subregion of the putamen [49].

### 3.3. Patient Phenotype?

In the same clinical studies, the severity of GID was not found to correlate with the severity of pretransplant L-dopa-induced dyskinesias (LID). However, a negative correlation was seen between the severity of postoperative “off-phase” GID and preoperative putaminal 18F-Dopa uptake [55, 57]. This finding indicates that the manifestation of “off-phase” dyskinesias after grafting, similar to that of L-dopa-induced “on-phase” dyskinesias [60, 61] might be related to the baseline severity of striatal dopaminergic denervation.

### 3.4. Tissue Storage?

In the Freed trial, cells were stored for up to 4 weeks before transplantation, In the Hagell series, the appearance of GID was reported in patients with grafts stored for 1–8 days. However, any hypothetical relationship between tissue storage and GID hypothesis was not supported by the Olanow trial, in which no grafts were stored for >48 hours.

### 3.5. Immunosuppression?

In the Olanow trial, the initial significant improvement in the grafted patients compared with sham-operated cases [6] was lost following withdrawal of immune suppression after 6 months. Also in two patients who came to autopsy, the grafts were surrounded by activated microglia suggesting an immune response [6]. Such inflammatory reactions could lead to reduced graft survival and functional deterioration [62, 63]. GIDs develop slowly over time and appear to be most pronounced in patients that have received no immune-suppression [5] or only mild immunosuppressive treatment [56]. There has been speculation, therefore, that such an ongoing inflammatory/immune process could affect the way the grafted DA neurons release and/or handle DA at the synaptic level, which in turn may constitute a triggering factor for the induction of dyskinesias [64]. 

### 3.6. Serotonergic Contamination?

It has now been shown that in 2 patients experiencing GID, there was excessive serotonergic innervation in their brains following transplant, (252–285% higher than comparable advanced PD patients). This was measured using functional imaging with ((11)C)-3-amino-4-(2-dimethylaminomethyl-phenylsulfanyl)  benzonitrile positron emission tomography (C^11^-DASB PET). Importantly this excessive serotonergic innervation was seen restricted to the areas of their grafts. There is mounting evidence that serotonergic neurons contaminating the original graft release dopamine in an uncontrolled manner and then lack the ability to reuptake DA and buffer extracellular DA levels leading to GID [65].

## 4. The Relationship between LID and GID

The clinical similarity between LID and GID suggests that similar pathophysiological mechanisms may underlie the development of both. Animal models strongly support the suggestion that the severity of LID in animals that have received putaminal grafts are related to the number of serotonergic neurons contained within the graft, as well as the severity of the dopaminergic lesion. Among animals receiving serotonin grafts [66], there was no impact on either motor asymmetry in the amphetamine-induced rotation test or spontaneous forelimb use in the cylinder test, but in contrast there was a progressive worsening of LID. Other studies have also confirmed that removal of serotonin innervation, or dampening of serotonin neuron activity by agonist drugs, results in a near complete blockade of LID in 6-OHDA lesioned rats [27].

It would appear that the relative abundance of dopaminergic compared to serotonergic neurons (whether host or grafted) is a critical factor in LID development after graft [67, 68]. It appears that, in the presence of a severe dopaminergic deficit, dopamine released from serotonergic neurons may trigger severe dyskinetic responses, but provided *∼*10–20% of the dopamine innervation remained intact, the grafted serotonin neurons have limited detrimental effect on dyskinesia severity. This has been corroborated independently showing that serotonergic neurons are not detrimental, provided sufficient dopamine neurons remain in the graft [69].

The appearance of LID after graft may occur via (i) dysregulated DA release from serotonergic neurons themselves, (ii) lack of reuptake of released DA due to insufficient DA neurons, (iii) inhibition of the dopamine transporter (DAT) on DA neurons by 5HT release thus preventing DA reuptake by DA neurons, and (iv) abnormal dopamine receptor supersensitivity (i.e., a postsynaptic component) as evidenced by increases in apomorphine-induced rotations [28, 68].

Despite these consistent findings of LID appearance following serotonergic contamination of cell grafts, “off-phase” dyskinesias (GID) do not appear to occur in animal models of PD undergoing cell grafts with the exception of very mild abnormal movements occurring in 2 studies [70, 71]. This is of major importance since there is little or no relationship between the change in LID following transplantation in patients (which tends to improve) and the development of GID. Given that the patients exhibiting GID are those that had the most severe dopaminergic deficits, in the absence of exogenous L-dopa administration, it seems likely that GID appearance must relate to dopamine production from the graft itself. Dopamine released into the extracellular space can be taken up by serotonergic neurons via the serotonin transporter and subsequently be rereleased as a false transmitter [72]. Furthermore, serotonin release can block the dopamine transporter and add to abnormal accumulation of dopamine in the synaptic cleft and onset of dyskinesia [65, 73]. Whether serotonergic neurons have a role in the development of “off-phase” GID simply by maintaining postsynaptic dopamine receptors in a supersensitive state has not yet been confirmed. Any relevance of abnormal synaptic plasticity as a secondary or parallel process in the development of GID has also yet to be comprehensively studied.

## 5. Gene Therapy for PD and Its Effects on Dyskinesia

Gene therapy represents an exciting new prospect for the treatment of patients with PD. This technology exploits the properties of viral vectors to invade host cells and incorporate DNA into the host genome. Appropriate modification of viral vectors allows control over which genes are incorporated and within PD research the main focus has been predominantly on genes encoding growth factors or enzymes involved in dopamine synthesis [74, 75]. Gene transfer offers a practical means of solving the problems associated with implanted hardware while still providing a continuous and selective delivery system of the desired gene/protein at the targeted site.

There are 4 gene therapy programs that have already reached the stage of clinical trial evaluation.

### 5.1. AAV2-Neurturin

AAV2-Neurturin, an analogue of glial-cell-derived neurotrophic factor (GDNF), has been developed in an attempt to provide trophic support to neurons/glia and thus manipulate the progression of PD. Both (GDNF) and Neurturin enhance dopaminergic neuron survival and nigrostriatal function in animal models of PD. Both factors provide protection from 6-OHDA-induced degeneration in rats [76] and provide neuroprotection in parkinsonian monkeys [77]. In a clinical trial of 58 patients at 12 months, delivery of neurturin to the striatum failed to show any change in the primary outcome measure (off-medication part III-UPDRS) [78]. There was not any change in dyskinesia scores in neither the Neurturin nor the sham surgery groups. Retrograde transport to the substantia nigra pars compacta (SNc) was lower (or possibly slower) than expected and therefore a follow-up evaluation is underway targeting both the striatum and SNc. It seems unlikely that this approach would lead to worsening of dyskinesia severity, indeed beneficial effects on dopaminergic number, survival or function should lead in theory to improvement in LID. Nevertheless, any disproportionate neurotrophic action on serotonergic neurons might in theory lead to provocation of dyskinesia, and these should be specifically sought during clinical evaluation of patients receiving this treatment.

### 5.2. AAV2-GAD

Gene therapy consisting of insertion of the glutamic acid decarboxylase gene (*GAD*) into the neurons of the STN offers an alternative therapeutic strategy. Inspired by the effectiveness of STN DBS, the hypothesis emerged that expression of GAD (the rate-limiting enzyme for GABA production) would inhibit overactivity in the STN and would improve off-medication UPDRS motor score. This strategy has been effective in the rat model of PD [79]. It is known that STN DBS can itself provoke “off-medication dyskinesia” but this is usually a transient phenomenon when it occurs and may be relieved by adjustment of the stimulation amplitude. Since the mechanism of action of STN DBS remains controversial, it remains theoretically possible that GAD gene therapy might also provoke “off-phase” dyskinesia. However in the results published to date in a double blind evaluation, no increase in dyskinesias was reported, while a modest improvement in PD severity was observed [80]. Open label follow up of these patients will allow further quantitative estimates of the extent to which LID may improve or deteriorate following this approach.

### 5.3. AAV-hAADC

Bilateral intraputaminal infusion of AAV-hAADC (adeno-associated virus-human aromatic L-amino acid decarboxylase), the enzyme that converts L-dopa into dopamine, aims to improve the conversion of exogenously administered L-dopa. Since this therapy remains dependent on exogenous administration of L-dopa, “off-phase” dyskinesia is unlikely to occur. However, striatal transfection with AAV-hAADC has been shown to increase LID in primate parkinsonian models if delivered in a nonhomogeneous way [81], reminiscent of the experience of “off-phase” graft-induced dyskinesia in cell therapy experiments. Despite these theoretical concerns, data published to date have shown an increase in on-time and reduction in off-time without any increase in LID [75]. Two patients had a transient increase in mild LID, but none experienced “troublesome LID,” which were reduced for the group as a whole [75].

### 5.4. LV-TH-GCH1-AADC-ProSavin

The ProSavin approach incorporates all 3 enzymes required for dopamine biosynthesis (tyrosine hydroxylase (TH), AADC, and GTP cyclohydrolase L1(GCH1)), with the aim of transfection of nondopaminergic cells so that they may produce and release dopamine endogenously. Again the theoretical concern is that transfected neurons may synthesize dopamine but may not be able to store and release this dopamine in a physiological manner, and an increase in dyskinesia may follow. The pilot phases of this program are using a dose escalation strategy, with careful evaluation of efficacy and dyskinesia severity at each stage, before proceeding with dose increases in subsequent patient cohorts. So far, there have been no concerns regarding “off-phase” dyskinesias. Further addition of the vesicular monoamine transporter 2 (VMAT2) gene to allow dopamine transport by transfected cells has not shown any advantage over the ProSavin approach in laboratory experiments [82].

An additional gene therapy program that has not yet reached clinical trial stage aims to deliver continuous dopamine therapy using AAV-TH-GCH1. By omitting the AADC enzyme, transfected cells would be able to synthesize L-dopa but rely on endogenous AADC activity to produce dopamine. This reduces the risk of uncontrolled dopamine production that cannot be stored or transported physiologically and aims to deliver “continuous dopaminergic stimulation (CDS),” to mimic the advantages observed using other methods of CDS [12]. In rodent models, this therapy has been shown to allow resistance to LID development [83].

All of the current gene therapy approaches, if successful, could permit a reduction of L-dopa dose and, therefore, achieve greater control of LID. No major concerns regarding “off-phase” dyskinesias have been reported among patients exposed to PD gene therapy. However, dose escalation to try and achieve greater efficacy from these approaches will necessitate continued vigilance with regard to dyskinesia emergence.

## 6. Preventing “Therapy-” Induced Dyskinesias

Our knowledge regarding the underlying causes of LID is growing, and it is clear that the pathophysiological processes are complex, depending on the number of intact dopaminergic terminals, the chronicity, and pattern of administration of L-dopa replacement as well as the possibility of genetic variability in pathways controlling receptor supersensitivity/internalization and synaptic plasticity. There is converging evidence to implicate nondopaminergic neurons, in particular serotonergic neurons releasing dopamine in a nonphysiological manner, as a major contributory factor in LID and also GID.

This knowledge is vital in trying to minimize the likelihood of “off-phase” GID developing in PD patients participating in future trials of fetal cell therapy. Careful attention must be paid to selecting the optimal patients phenotype with respect to the severity of their PD and by implication the number of surviving dopaminergic terminals at the time of transplantation. Patients with advanced dopaminergic degeneration will be at greater risk of developing dyskinesia from grafts that contain an excessive number of serotonergic neurons. Patients with less advanced dopaminergic cells loss should be more tolerant of a greater number of serotonin contaminating cells. The relative extent of dopamine/serotonin striatal innervations can be revealed using preoperative functional imaging, to quantify the extent and severity of both dopaminergic and serotonergic innervations in the striatum.

The perioperative and intraoperative details are also extremely important. It is possible to manipulate the number of contaminating serotonergic neurons within grafts, through optimisation of the dissection margins in the ventral mesencephalon of the fetal tissue, during graft harvesting. Furthermore, clinical and animal data both suggest that surgical targeting should avoid the ventral putamen. While the type and duration of immunosuppressive regimes may or may not have major relevance for GID development, it is likely that overall cell survival is improved by maintaining immunosuppression for longer than the 6 months adopted in previous cell therapy trials.

### 6.1. Additional Use of Nondopaminergic Medications

The use of serotonin (5HT-1A) agonists as antidyskinetic agents is not new. Buspirone, a (5-hydroxytryptamine (5HT 1A) receptor agonist, has previously showed beneficial effects lessening the severity of LID [84], by dampening transmitter release from serotonergic neurons through activation of inhibitory 5HT-1A autoreceptors without any worsening of extrapyramidal symptoms. Beneficial effects seen among patients with GID have also been reported [65]. Sarizotan (also a 5-HT1A receptor agonist but with additional high affinity for D3 and D4 receptors) showed encouraging results in an open-label evaluation [85]; however, in a blinded trial the effects of Sarizotan on LID were disappointing [86]. While the mechanism(s) remain unclear, one likely explanation for the possible beneficial effect of serotonin receptor agonists on LID is that stimulating 5HT1A receptors diminish dysregulated release of dopamine from raphe-striatal serotonergic neurons. However, it has also been shown that 5HT1A agonist drugs alleviate dyskinesias provoked by direct D1 receptor agonists, suggesting a further interaction between D1 and 5-HT1A receptors [87].

The beneficial effects of Amantadine on LID through its action on NMDA receptors have prompted further study of agents acting on the corticostriatal glutamatergic input evaluating the effects of metabotropic glutamate receptor (mGLUR) antagonists and Adenosine A2A receptor antagonists. AFQ056 is a potent, selective mGLUR5 antagonist that shows antidyskinetic effects in a rodent PD model and has been shown to have significant antidyskinetic effects in 2 small blinded trials [88]. Istradefylline (an A2A antagonist) has been shown to improve motor function and reduce the development of LID in nonhuman primates [89]; indeed rodent A2a knockout animals do not develop LID [90]. In patients with PD, however, multiple randomised trials have failed to show beneficial effects of Istradefylline on LID severity [80, 91]. *α*2Adrenergic agonists modulate the activity of the direct striatopallidal pathway and have been shown to reduce LID in PD patients but their use in PD patients has been limited by development of side effects [92, 93]. Given the demonstration of beneficial effects in animal models [36, 37], a further emerging possibility might be the use of D3 antagonists to allow D1 receptor internalisation, with the aim of reducing the postsynaptic “supersensitive” state [94]; however, this approach has yet to be evaluated in patients.

## 7. Conclusions

The current and future gene therapy and cell therapy programs represent a great source of optimism for patients with PD. However, PD is a heterogeneous disease and only a subset of patients are likely to benefit—perhaps the subgroup of patients with young onset PD that remain with a predominantly motor deficit for many years [95]. In these individuals, LID is a major problem and the success of cell or gene therapy will depend on providing relief of PD “off” symptoms without “off-phase”/therapy-induced dyskinesia.

Dyskinesia development has multiple determinants, and therefore multiple potential opportunities exist to intervene and prevent their occurrence. Some of these processes may be relevant solely as a secondary consequence of nonphysiological dopamine release (presynaptic component) from serotonergic or other neurons, or as a downstream effect of chronic dopamine receptor supersensitivity (postsynaptic component). Consequent downstream changes in synaptic plasticity may account for abnormal oscillatory firing patterns, throughout the basal ganglia circuitry. L-dopa itself (presumably when released physiologically) has been identified as having a role in the restoration of normal synaptic plasticity, as evidenced from recordings of patients undergoing high-frequency stimulation of the substantia nigra pars reticulata, in both on- and off-medication states [96, 97]. Whether other pharmacological options such as Buspirone, AFQ056, or D3 receptor antagonists can be exploited to relieve off-medication GID needs further study.

In parallel with approaches to relieve LID and GID, our understanding of the importance of the ratio of serotonin and dopaminergic neurons allows optimisation of future interventions and accompanying trial design. Our greater understanding of the causes of dyskinesia, either L-dopa related or graft and gene therapy induced, represents a considerable step towards ensuring the success of future gene and cell therapy programs.
